# Piezo1 agonists are potent off-target inhibitors of the Kv1.3 channel via gating modification

**DOI:** 10.1016/j.molpha.2026.100149

**Published:** 2026-07-29

**Authors:** Kyan Huang, Heesung Shim, Heike Wulff, Hai M. Nguyen

**Affiliations:** 1 Department of Pharmacology, School of Medicine, University of California, Davis, Davis, California; 2 Physical and Life Sciences, Lawrence Livermore National Laboratory, Livermore, California

**Keywords:** Kv1.3, Piezo1, Ion channel pharmacology, Ca^2+^ signaling, Inflammation, Molecular docking

## Abstract

The mechanosensitive Piezo1 channel is crucial for the regulation of calcium (Ca^2+^) signaling within myeloid tissue macrophages. However, the current lack of comprehensive pharmacological characterization for existing Piezo1 agonists complicates the assessment of Piezo1 agonism as a viable therapeutic strategy. We report inhibition of the voltage-gated Kv1.3 potassium (K^+^) channel as a critical off-target effect of the most widely used Piezo1 agonists, Yoda1 and Yoda2. Kv1.3 is essential for the activation and innate immune functions of tissue macrophages. Patch-clamp electrophysiological experiments demonstrate that Yoda1 (IC_50_ = 9.0 *μ*M) and Yoda2 (IC_50_ = 5.9 *μ*M) state-independently inhibit Kv1.3 by accelerating channel inactivation. Functionally, blockade of Kv1.3 by Yoda2 in Piezo1-deficient bone marrow–derived macrophages depolarizes membrane potential and reduces store-operated calcium entry and interleukin-1*β* release. Using RosettaLigand docking and molecular dynamics simulations, we identified a network of key residues near the Kv1.3 outer pore as the potential binding site. Our results suggest that Kv1.3 blockade likely contributes substantially to the reported anti-inflammatory effects of Yoda1 and Yoda2. This provides a plausible mechanism that aligns with the established principle that reducing Ca^2+^ signaling is anti-inflammatory, while helping to reconcile existing literature discrepancies regarding Piezo1 activation.

**Significance Statement:**

Targeting Ca^2+^ channels such as Piezo1 is vital for controlling chronic inflammation, but selectivity is challenging. This study reveals that the best-known Piezo1 agonists exhibit a critical off-target effect by blocking the Kv1.3 channel, which is essential for macrophage and microglial immune function. This concurrent inhibition reduces inflammatory Ca^2+^ signaling and interleukin-1*β* secretion. This work underscores the necessity for rigorous selectivity screening of Piezo1 drugs and establishes Kv1.3 inactivation modification as a viable strategy for designing novel anti-inflammatory therapeutics.

## 1 Introduction

As professional responders to pathogen-associated molecular patterns and damage-associated molecular patterns, myeloid tissue macrophages, including the central nervous system–resident microglia, depend on intracellular calcium (Ca^2+^) conducted by various ion channels such as Ca^2+^-release-activated Ca^2+^ (CRAC) channels and the purinergic purinergic type 2 ionotropic (P2X) receptors for proliferation, migration, and cytokine production.^1^ Tissue macrophages also actively respond to mechanically distinct microenvironments,^2^ in part, because of the abundant expression of the mechanosensitive channel Piezo1.^3^, ^4^, ^5^, ^6^, ^7^

While Piezo1 activation alone does not polarize macrophages or microglia, both physical and chemical activation by Yoda1, a small-molecule agonist of Piezo1,^8^^,^^9^ have been shown to enhance proinflammatory responses of these cells to classical stimuli such as lipopolysaccharide (LPS) and interferon gamma.^7^^,^^10^, ^11^, ^12^ Accordingly, Piezo1 deficiency or pharmacological inhibition (eg, by grammostola spatulata mechanotoxin 4 [GsMTx4]) consistently reduced Nod-like receptor protein 3 or nuclear factor *κ*B (NF-*κ*B) activation, suppressed interleukin (IL)-1*β*, IL-6, and tumor necrosis factor *α* (TNF-*α*) release in LPS-treated cells, and inhibited the progression of chronic inflammatory diseases such as inflammatory bowel disease.^7^^,^^12^^,^^13^

However, several studies also showed that Yoda1 reduced inflammation. Malko et al^11^ reported that Yoda1 inhibited the production of IL-6 and TNF-*α* in LPS-primed microglia and BV2 cells by blocking LPS-induced Ca^2+^-dependent activation of NF-*κ*B. Similarly, pretreatment of RAW264.7 and bone marrow–derived macrophages (BMDM) with Yoda1 inhibited the secretion of proinflammatory factors induced by LPS, while promoting reactive oxygen species accumulation and macrophage apoptosis.^14^ Furthermore, in a microfluidic model of the human Alzheimer disease (AD) neuroglial microenvironment, Piezo1 activation reduced both microglial and astrocytic inflammation, amyloid *β* (A*β*) deposition, and tau hyperphosphorylation, thereby alleviating synaptic impairment and neuronal loss.^15^ Consequently, the therapeutic potential of pharmacological Piezo1 activation is currently uncertain, given the conflicting reported effects of the agonist Yoda1.

Immune cell Ca^2+^ influx is tightly regulated by the electrochemical gradient across the cell membrane.^16^^,^^17^ A key determinant of the resting membrane potential (RMP) in macrophages and microglia is the voltage-gated K^+^ channel Kv1.3,^18^^,^^19^ which has been shown to be upregulated by stimulation with LPS and in AD models of A*β* pathology.^20^^,^^21^ K^+^ efflux conducted by Kv1.3 maintains a negative membrane potential, which is essential for sustaining the Ca^2+^ driving force and proper intracellular signaling.^22^ Here, we report that both Yoda1 and its derivative Yoda2 state-independently block Kv1.3 channels with low-micromolar potency and alter their biophysical properties. In functional assays, Yoda2 reduces inflammatory calcium signaling and IL-1*β* secretion in macrophages. Taken together, our data demonstrate that Piezo1 agonists exert an off-target inhibition of Kv1.3, a mechanism that likely substantially contributed to their observed anti-inflammatory effects and reconciles existing conflicts regarding Piezo1’s pharmacological potential.

## 2 Materials and methods

### 2.1 Cell culture and expression

For stable expression, L929-mKv1.3 (developed in-house) and murine erythroleukemia–hKv1.5 cells (obtained from Dr. E. Conley, University of Leicester, UK) were maintained in growth medium supplemented with 100 mg/mL geneticin (G418, Sigma-Aldrich) and plated on poly-L-lysine-coated (Sigma-Aldrich) coverslips 24 hours prior to recording. For transient expression, CHO cells (American Type Culture Collection #CCL-61) were cultured in Dulbecco’s modified Eagle’s medium supplemented with 10% FBS (Gibco) and 1% penicillin/streptomycin (Sigma-Aldrich) at 37 °C and 5% CO_2_ (Airgas). Cells were transfected with wild-type (WT) or mutant human Kv1.1, Kv1.2, or Kv1.3 using FuGene 6 (Promega). Nontagged constructs were cotransfected with a plasmid encoding the Enhanced Green Fluorescent Protein C1 (pEGFP-C1, Invitrogen) at 3:1 ratio to identify transfected cells; EGFP-positive cells were recorded 24–48 hours post-transfection and approximately 1 hour after reseeding onto poly-L-lysine-coated coverslips.

The N-terminally truncated hKv1.3 construct (L23499.1, missing residues 1–52) in the plasmid recombinant construct/cytomegalovirus (pRc/CMV) was a gift from Dr Carol Deutsch (University of Pennsylvania). Although this truncated cDNA was used in all Kv1.3 experiments, we used the full-length hKv1.3 numbering system throughout this article for clarity. For example, residue H399 in the truncated construct is referred to as H451, such that the H399V mutation is labeled H451V. Mutants were generated via site-directed mutagenesis (QuikChange II XL, Agilent) and verified by nanopore sequencing (Plasmidsaurus). Additionally, turbo Green Fluorescent Protein (tGFP)-tagged hKv1.1 (NM_000217) and myelocytomatosis - aspartate-tyrosine-lysine-aspartate-aspartate-aspartate-aspartate-lysine (Myc-DDK)-tagged hKv1.2 (NM_004974) were obtained from OriGene Technologies.

### 2.2 Reagents

Pharmacological agents, including Yoda1 (#5586), Yoda2 (#8051), Dooku1 (#6568), and thapsigargin (#1138), were purchased from Tocris Bioscience. The Kv1.3-selective inhibitor 5-(4-phenoxybutoxy)psoralen (PAP-1) was synthesized in-house as previously described.^23^

### 2.3 Tissue macrophage isolation and differentiation

Primary tissue macrophages were isolated from enzymatically digested ventricular tissues of 10–12-week-old male C57BL/6J mice^24^ using CD11b^+^ immunomagnetic beads (#130-049-601, Miltenyi Biotec). Following isolation, cells were stimulated with LPS (200 ng/mL) as previously described.^25^

BMDMs were differentiated from bone marrow progenitors of CX3CR1-S6f-Pz1LoF (Piezo1 knockout [KO]) and CX3CR1-S6f (Control) mice (kindly gifted from Dr Shivashankar Othy, University of California, Irvine).^7^ These mice express Salsa6f, a genetically encoded ratiometric Ca^2+^ indicator comprising tdTomato GCaMP6f by a V5 epitope tag (tdTomato-V5-GCaMP6f).^26^ To drive differentiation, bone marrow cells were cultured in the presence of macrophage colony-stimulating factor (MCSF) and granulocyte-MCSF (Gibco) for 7–8 days.^27^

### 2.4 Electrophysiology

#### 2.4.1 Recording conditions and compound preparation

Whole-cell patch-clamp recordings were performed at 22–24 °C using an EPC-10 USB amplifier and PatchMaster software (HEKA Elektronik). Standard Ringer’s bath solution (prepared in-house) contained (in mM): 160 NaCl, 4.5 KCl, 2 CaCl2, 1 MgCl2, and 10 HEPES (pH 7.4, 300 mOsm). Pipettes had resistances of 2–4 MOhm. Internal solutions (300 mOsm, pH 7.2) contained 10 mM HEPES (Sigma-Aldrich) and 2 mM MgCl_2_ (_Sigma-Aldrich_). For current-clamp experiments, the internal solution was supplemented with 160 mM KCl and 10 mM EGTA. For voltage-clamp experiments, the internal solution contained 145 mM potassium flouride (KF) and either 10 mM EGTA or 10 mM 1,2-bis(2-aminophenoxy)ethane- N,N,N',N'-tetraacetic acid (BAPTA).

Yoda1 and Yoda2 (prepared as 10 mM in 100% DMSO [Sigma-Aldrich] stocks) were diluted into the bath solution to final working concentrations (0.001%–0.1% DMSO) immediately before the experiment. Solubility was verified via visual inspection. Yoda2 remained stable up to 100 *μ*M, whereas Yoda1 showed visible precipitation above 10 *μ*M, consistent with the reported ∼160-fold higher aqueous solubility of Yoda2 compared with Yoda1.^28^

#### 2.4.2 Data acquisition and kinetic analysis

Voltage-clamp recordings began 5–10 minutes after break-in to ensure stability. Cells were held at −90 mV and pulsed to +40 mV for 200 milliseconds every 10–30 seconds. Because of the acceleration of inactivation by Yoda1/Yoda2, inhibitory potency was quantified using the fractional area under the curve. Concentration-response curves from individual cells were fitted with the Hill equation: Fractional area under the curve = 1 / (1 + ([inhibitor]/IC_50_)^nH^).

Kinetic parameters, including onset of inhibition, deactivation (tail current decay), and inactivation time constants, were determined by fitting to a single exponential function: y = y0 + A·e^−t/*τ*^. Recovery from inactivation was fitted to y = 1 − a·e^−x/b^, where b represents the recovery time course. Activation kinetics (10%–100%I_max_) were fitted to y = A(1 − e^−(t−t0)*τ*^).

#### 2.4.3 Voltage dependence

Voltage dependence of activation was assessed using steps from −80 to +60 mV. Conductance (G) was calculated as I / (V − Vr) and normalized to fit the 2-state Boltzmann equation: G/Gmax = [1 + e^−0.03937·z·(v−v^_1/2_)]^−1^. Steady-state inactivation was assessed with a 5-second conditioning prepulse (−120–0 mV); normalized peak currents were fitted to I/Imax = [1 + e^(v−v^_1/2inact_)^/k^]^−1^.

#### 2.4.4 Current-clamp

RMP and responses to 2-second depolarizing current ramps (0–50 pA) were recorded in current-clamp mode. Data are presented as mean ± SD, except for IC_50_ values, which are reported as mean with 95% confidence intervals (CIs). The number of independent observations (*n*) refers to individual cells collected from *N* ≥ 2 independent cultures.

### 2.5 Statistics

Statistical analyses were performed using GraphPad Prism software (GraphPad Software, LLC). The choices of specific statistical tests, error bars, and sample sizes (*n* cells, *N* independent cultures) are detailed in the respective figure legends. Differences were considered statistically significant when *P* < .05.

### 2.6 Molecular docking

Ligand docking was performed using RosettaLigand (Rosetta version 2021.16).^29^ Ligand conformers were generated using Molecular Operating Environment (Chemical Computing Group 2024), and Rosetta’s molfile_to_params.py script was used to produce the corresponding parameter files. The docking protocol consisted of (1) low-resolution rigid body perturbation of the ligand (Transform mover), (2) high-resolution refinement with side-chain repacking and limited backbone flexibility of residues within 6–7Å of the ligand (HighResDocker and MoveMapBuilder), and (3) final full-atom minimization using the standard ligand score function. A softened repulsive potential (ligand_soft_rep) was applied during the initial high-resolution search, followed by refinement with the full-atom ligand score function derived from ref2015.wts. Hydrogen bonding, electrostatics, and van der Waals interactions were evaluated using the corresponding Rosetta energy terms. A total of 10,000 docking models were generated for each ligand. Docked poses were ranked based on the interface score (interface_delta_X), and the 10 lowest-energy models were selected for detailed structural analysis.

### 2.7 Molecular dynamics simulations

The Kv1.3 protein–ligand system was constructed using Chemistry at HARvard Macromolecular Mechanics - Graphical User Interface (CHARMM-GUI, Lehigh University, Bethlehem, PA) with the protein embedded in a 1-palmitoyl-2-oleoylphosphatidylcholine lipid bilayer (POPC), defined by its force field topology parameterization for simulation. The system was solvated with Transferable Intermolecular Potential 3-Point (TIP3P) water molecules and 0.15 M KCl. The CHARMM36m force field was used for the protein and lipids, and ligand parameters were generated using the CHARMM General Force Field (University of Maryland). Molecular dynamics (MD) simulations were performed using GROMACS (Uppsala University/Stockholm University, Sweden) under periodic boundary conditions. Long-range electrostatic interactions were treated using the Particle Mesh Ewald method. The system was equilibrated following energy minimization and equilibration steps, and production simulations were carried out for 100 ns. All simulations were performed in the isothermal–isobaric ensemble with constant number of particles, pressure, and temperature (NPT) at 303.15 K. Four independent replicates were conducted for each system.

### 2.8 Ca^2+^ time-lapse imaging and cytokine assays

Real-time changes in intracellular Ca^2+^ concentration ([Ca^2+^]_i_) were measured in BMDMs seeded onto glass coverslips precoated with 0.01% poly-L-lysine at a density of 10,000–15,000 cells/cm^2^. The Ca^2+^ signals were recorded using a BZ-X780 fluorescence microscope (Keyence LLC) equipped with a time-lapse imaging module. The GCaMP6f signal was monitored at Ex/Em 495/519 nm as previously described.^22^ To evaluate the effect of Yoda2 inhibition on [Ca^2+^]_i_ changes, cells were preincubated with 5 *μ*M Yoda2 30 minutes before and throughout the course of the recording duration. Fluorescence measurements were acquired at 2-second intervals at room temperature. To ensure single-cell resolution and avoid possible signaling from coupled cells, only subconfluent areas of the coverslips were selected for analysis. Image data were analyzed off-line with Fiji (ImageJ) with the Region of Interest 1 (ROI-1) click tool.^30^^,^^31^ Changes in [Ca^2+^]_i_ are represented as ΔF/F_0_, where ΔF is the baseline-subtracted change in fluorescence, and F_0_ is the initial baseline intensity.

Cytokine ELISA assays were performed on Piezo1 KO BMDMs seeded in 24-well plates at a density of 250,000 cells/well in Dulbecco’s modified Eagle’s medium supplemented with 5% FBS. Prior to treatment, cells were serum-starved for 24 hours. Following a 24-hour stimulation with 300 ng/mL LPS, IL-1*β* concentrations in the culture supernatants were quantified using ELISA kits (R&D Systems) according to the manufacturer’s protocol. Assays were conducted using *N* = 3 independent biological replicates (derived from 3 different cell cultures), with each treatment condition performed in *n* = 3 technical triplicates (3 wells per condition per experiment).

## 3 Results

### 3.1 Piezo1 agonists inhibit Kv1.3 channels

Because the apparent reported anti-inflammatory effect of the Piezo1 agonist mirrors that of Kv1.3 inhibitors, we first investigated its potential action on heterologously expressed Kv1.3 channels using whole-cell patch-clamp electrophysiology. We found that Yoda1 inhibits recombinantly expressed mouse Kv1.3 channels and enhanced current inactivation, resulting in reduced current amplitude at the end of the 200-ms depolarizing pulse (Fig. 1A). The IC_50_ of Yoda1 against mKv1.3, calculated from the area (total anodic charge Q) under the curve to account for changes in current kinetics, is 9.0 *μ*M (95% CI, 7.9–10.2 *μ*M) with a corresponding Hill coefficient (*nH*) of 2.2 (95% CI, 1.6–2.8) (Fig. 1D).

**Fig. 1:**
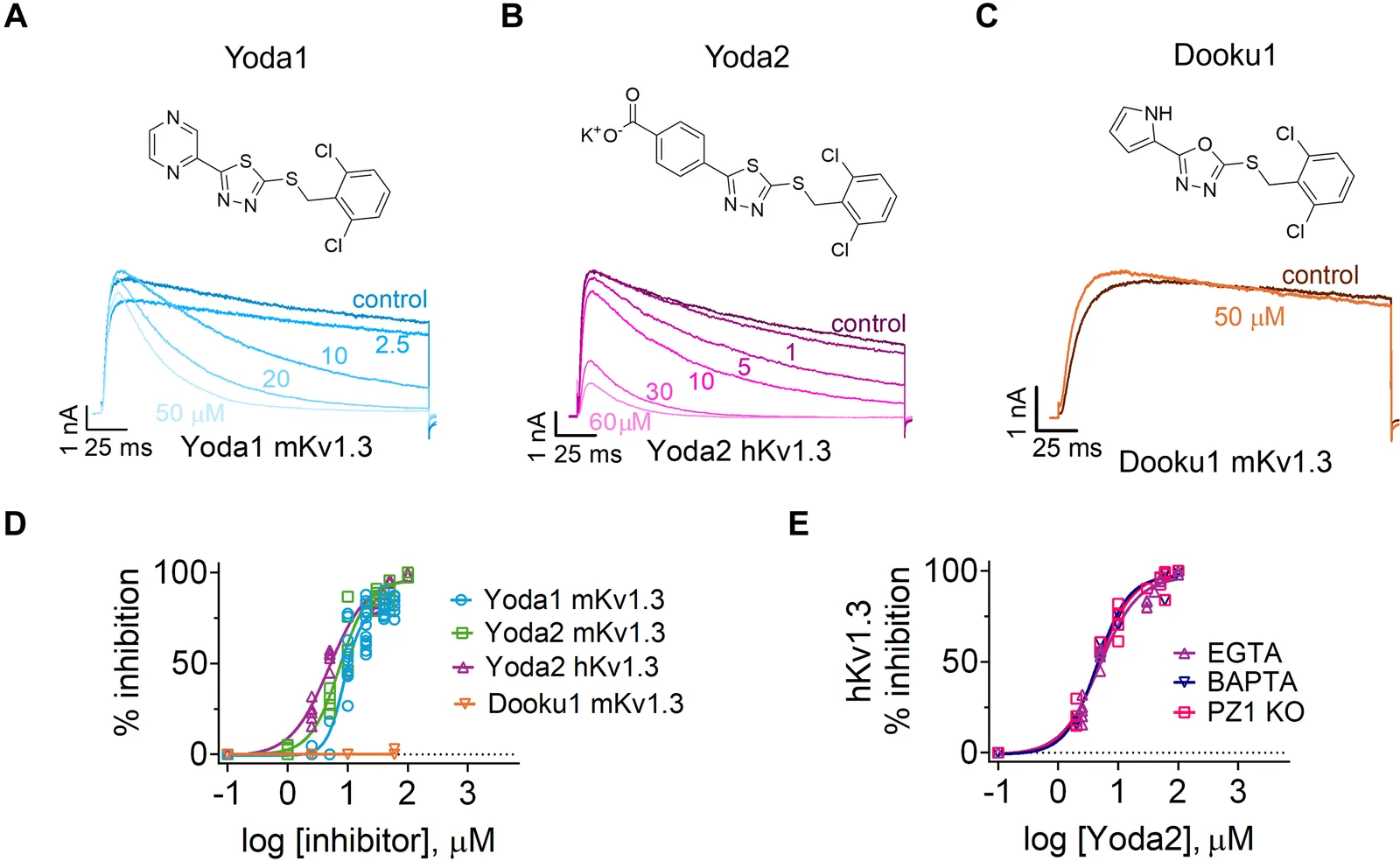
Pharmacological inhibition of Kv1.3 by Piezo1 modulators. (A–C) Chemical structures and representative traces showing inhibition of mKv1.3 by Yoda1 (A), hKv1.3 by Yoda2 (B), and mKv1.3 with 50 *μ*M Dooku1 (C). (D) Concentration-response curves for Piezo1 modulators on Kv1.3. IC_50_ values (mean, 95% CI): Yoda1 (mKv1.3), 9.0 *μ*M (7.9–10.2); Yoda2 (hKv1.3), 5.9 *μ*M (5.0–8.6); Yoda2 (mKv1.3), 7.7 *μ*M (6.8–9.0); Dooku1 (mKv1.3), >50 *μ*M (nonblocking). (E) Kv1.3 inhibition by Yoda2 across experimental conditions (IC_50_, 95% CI): EGTA internal, 5.9 *μ*M (5.0–8.6); BAPTA internal, 4.6 *μ*M (3.9–5.6); and Piezo1 KO HEK-293 cells, 4.9 *μ*M (4.2–5.6). IC_50_ values were determined from fractional area under the curve fits. Data represent the mean of *n* = 3–6 cells from *N* ≥2 independent cultures. Each cell was exposed to ≥3 incrementing concentrations.

Because of its high lipophilicity, Yoda1 exhibits poor solubility at concentrations of 20 *μ*M and above, limiting further investigation at higher concentrations. Yoda2, a more soluble derivative of Yoda1, was synthesized by replacing the pyrazine ring of Yoda1 with a 4-benzoic acid moiety.^28^ We tested Yoda2 against mouse and human Kv1.3 channels. Yoda2 exhibited slightly better potency and reduced variability than Yoda1, consistent with its enhanced solubility at maximal test concentrations. Against hKv1.3, Yoda2 inhibited currents with an IC_50_ of 5.9 *μ*M (95% CI, 5.0–8.6 *μ*M) and *nH* of 1.5 (95% CI, 0.9–2.3). Similarly, against mKv1.3, it displayed an mKv1.3 of 7.7 *μ*M (95% CI, 6.8–9.0 *μ*M) and an *nH* of 2.1 (95% CI, 1.6–3.0) (Fig. 1, B and D).

We further tested Dooku1, a structural analog and competitive antagonist of Yoda1, on Piezo1.^32^ In Dooku1, replacement of Yoda1’s pyrazine ring and thiadiazole with a pyrrol-2-yl group and an oxadiazole resulted in a compound binding to the same binding site and preventing Yoda1-induced Piezo1 activation, without activating the channel itself.^9^^,^^32^ Bath applications of up to 50 *μ*M Dooku1 showed no Kv1.3 blocking activity (Fig. 1C). Furthermore, preincubation of hKv1.3 with Dooku1 did not prevent the activity of Yoda2 (Supplemental Fig. 1), suggesting that Dooku1 does not interact with hKv1.3 in a manner that prevents Yoda2’s effect. Overall, these data suggest that Yoda1 and Yoda2 inhibit Kv1.3 with a potency similar to their reported effectiveness in sensitizing Piezo1 to mechanical activation. To rule out the involvement of Ca^2+^-mediated or Piezo1-mediated indirect inhibition of Kv1.3, we repeated our pharmacological characterization of Yoda2 using an intracellular solution containing 10 mM BAPTA as well as in Piezo1 KO HEK-293 cells. The potency of Yoda2 against hKv1.3 when recorded with BAPTA-buffered intracellular solution (IC_50_ = 4.6 *μ*M, 95% CI, 3.9–5.6 *μ*M; *nH* of 1.8, 95% CI, 1.4–2.3) and in Piezo1 KO HEK-293 cells (IC_50_ = 4.9 *μ*M, 95% CI, 4.2–5.6 *μ*M; *nH* of 1.6, 95% CI, 1.4–1.9) are comparable to that in EGTA-containing internal solution (Fig. 1E).

### 3.2 Yoda2 accelerates Kv1.3 gating kinetics and shifts the voltage dependence

Given the pronounced effects of Yoda1 and Yoda2 on Kv1.3’s inactivation in addition to current block (Figs. 1, B and C, 2, A and B), we proceeded with a detailed mechanistic analysis of Yoda2’s activity on Kv1.3. We first investigated Yoda2’s effect on the kinetics of Kv1.3 currents. Yoda2 enhanced both Kv1.3 activation (Fig. 2, C and D) and inactivation kinetics (Fig. 2E) when compared with control (*P* < .001). The voltage dependence of activation and inactivation was also shifted in the presence of Yoda2. Specifically, Yoda2 shifted the V_1/2_ of activation (Fig. 2F) in the depolarized direction by 12.5 mV (control: −24.3 ± 4.0 mV, *n* = 9; Yoda2: −11.8 ± 2.8 mV, *n* = 16; *P* < .01). Interestingly, Yoda 2 also significantly increased the apparent gating charge *z* from 2.4 ± 0.8 (*n* = 9) to 4.8 ± 1.1 (*n* = 16, *P* < .01), suggesting that Yoda2 increases the voltage-sensitivity of the activation process, forcing a more concerted voltage-sensor translocation.

**Fig. 2:**
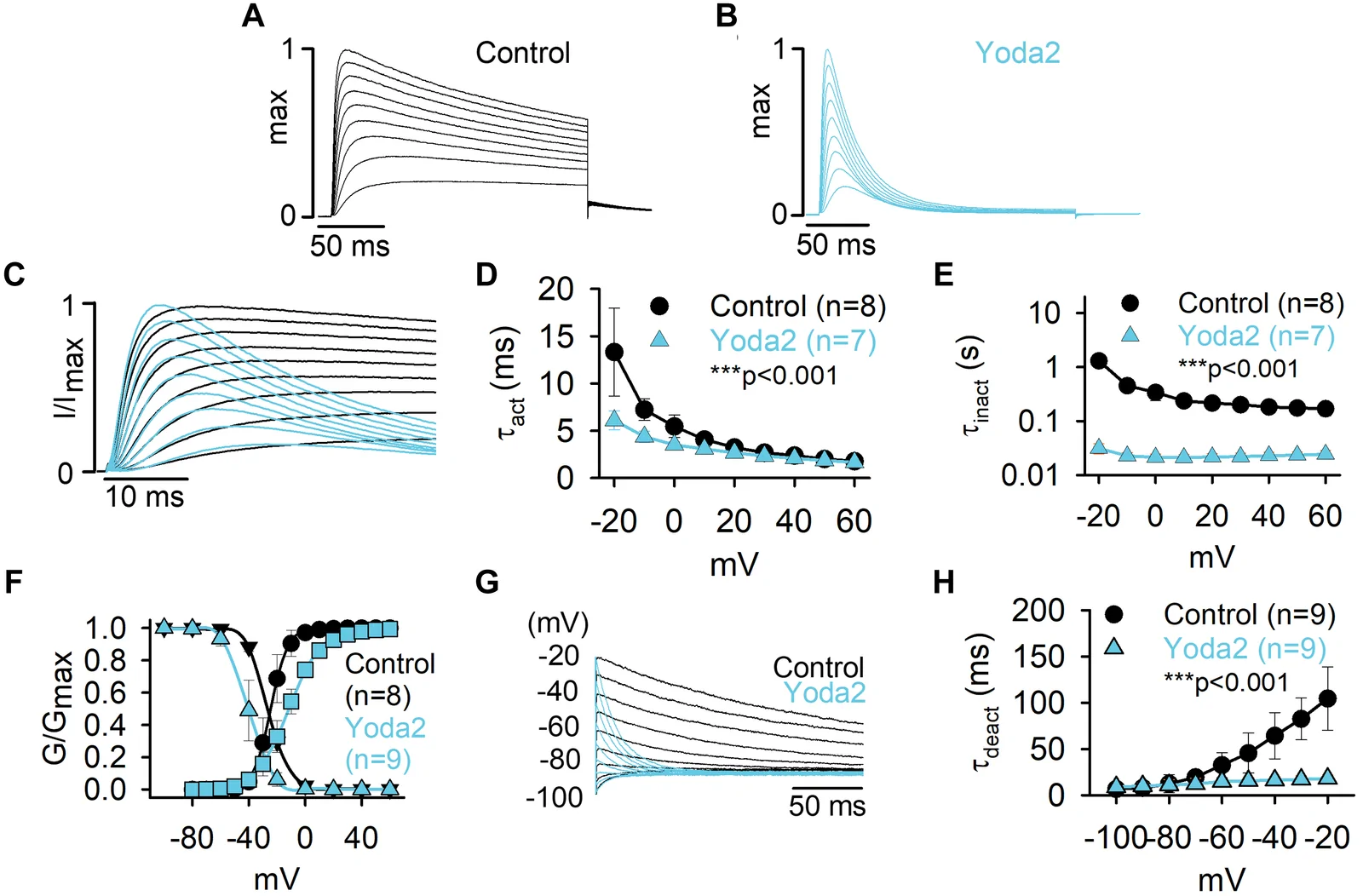
Kinetic and voltage-dependent modulation of hKv1.3 by Yoda2. (A, B) Representative normalized traces in control (A) and 30 *μ*M Yoda2 (B). (C) Trace overlay illustrating accelerated kinetics with Yoda2. (D, E) Quantified activation (D), activation and inactivation (E) time constants (*τ*) across voltages. (F) Voltage dependence of activation (G-V) and steady-state inactivation curves. (G–H) Representative deactivation current overlay (G) and quantified deactivation time constants (H). Data presented as mean ± SD of *n* cells from *N* ≥2 independent cultures. Statistical significance (∗∗∗*P* < .001) at individual voltages was determined by an ordinary two-way ANOVA followed by Šidák multiple comparisons post hoc test (panels D, E, and H) or nested *t* test (panel F).

Furthermore, Yoda2 shifted the V_1/2_ of inactivation in the hyperpolarized direction by 14.2 mV (control: −26.0 ± 1.8 mV, *n* = 7; Yoda2: −40.2 ± 5.4 mV, *n* = 11; *P* < .01). However, Yoda2 did not alter the inactivation slope factor *k* (control: 6.9 ± 0.2, *n* = 7; Yoda2: 6.8 ± 0.3, *n* = 11; *P* = .83), indicating that, although the drug stabilizes the inactivated state at more negative membrane potentials, it does not modify the fundamental cooperativity or apparent charge movement coupled to the inactivation gate. Finally, we observed an enhancement of deactivation of Kv1.3 by Yoda2 (Fig. 2, G and H; *P* < .001), suggesting that Kv1.3 channels close faster in the presence of Yoda2 than under control conditions.

### 3.3 State-dependency and access of Yoda2 inhibition

To determine if Yoda2 inhibition is state-dependent, we performed resting preincubation experiments. Yoda2 exerted near-maximal inhibition of Kv1.3 upon the very first depolarization pulse after a 3-minute rest at −90 mV (Fig. 3A), demonstrating that the compound accesses its binding site while the channel is closed. Inhibition was largely saturated at the first pulse and did not increase with repetitive pulsing (Fig. 3B), indicating a lack of use dependence.

**Fig. 3:**
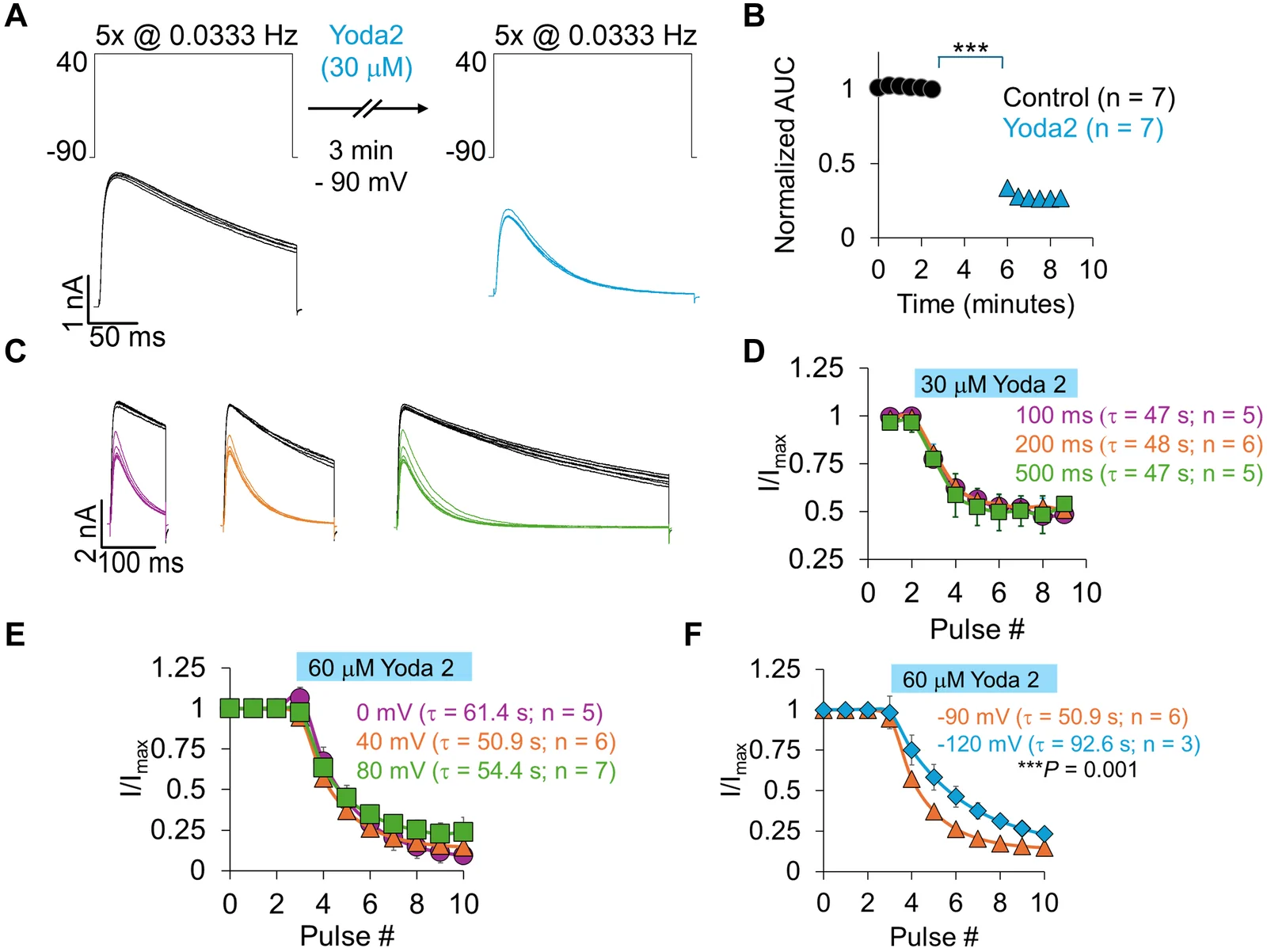
Yoda2-mediated inhibition of Kv1.3 is state-independent. (A, B) Protocol schematic and traces (A) and normalized total charge (area under the curve [AUC]) quantifying resting-state inhibition (paired *t* test; *n* = 7; ∗∗∗*P* < .001) (B). (C–E) Representative traces (C) and normalized peak currents demonstrating inhibition is independent of pulse duration (D) or depolarization voltage (E) (one-way ANOVA with Tukey post hoc test; *n* = 5–7; *P* > .05). (F) Normalized peak currents showing inhibition is dependent on holding potential (unpaired *t* test; *n* = 5–7). All data represent mean ± SD from *N* ≥2 independent cultures.

Further kinetic analysis showed that the time constants for block development (*τ*_block_) were nearly identical across pulse durations of 100, 200, and 500 milliseconds: 47.0 ± 10.8, 48.3 ± 6.5, and 47.2 ± 3.4 seconds, respectively (Fig. 3, C and D). Because increased occupancy of the open and inactivated state did not accelerate the rate of block, these results confirm that binding is driven by diffusion to the resting channel rather than state-specific transitions during the pulse. We also revealed that the inhibition rate was independent of depolarization voltage (0, 40, or 80 mV) (Fig. 3E), further suggesting that access is not coupled to voltage-sensor movement or pore opening. This distinguishes Yoda2 from inhibitors such as PAP-1, which preferentially bind to the inactivated state and require multiple pulses to reach equilibrium.^23^

However, we found that steady-state inhibition is sensitive to the resting configuration. Decreasing the holding potential from −90 mV to −120 mV, shifting channels into a deeper resting state, increased the time-to-steady-state block (Fig. 3F). This suggests that, although access to the closed channel is state-independent, Yoda2’s association rate or affinity is modulated by the resting state, with a preference for potentials near −90 mV.

### 3.4 Yoda2 promotes use dependence but does not affect recovery from inactivation

Because Yoda2 accelerated C-type inactivation, we investigated its impact on inactivation-induced use-dependent accumulation and recovery from inactivation. C-type inactivation is a slower, cumulative form of channel inactivation, distinct from N-type, often associated with conformational changes in the channel’s outer pore or selectivity filter.^33^^,^^34^ Repetitive depolarization at frequencies such as 1 Hz typically leads to use-dependent current rundown, a phenomenon attributed to the channel’s inability to fully recover from C-type inactivation between pulses. We observed a small but significant acceleration of use-dependent current rundown in the presence of Yoda2 (Fig. 4, A and B; *P* < .05). The overall availability of Kv1.3 for subsequent activation by depolarization pulses is critically determined by its rate of recovery from inactivation. Interestingly, despite enhancing inactivation, Yoda2 did not affect the rate at which Kv1.3 recovers from inactivation (Fig. 4, C and D). Taken together, these findings suggest that Yoda2 affects Kv1.3’s overall activity by accelerating both channel closing and inactivation but does not prolong the duration the channel remains in the inactivated state.

**Fig. 4:**
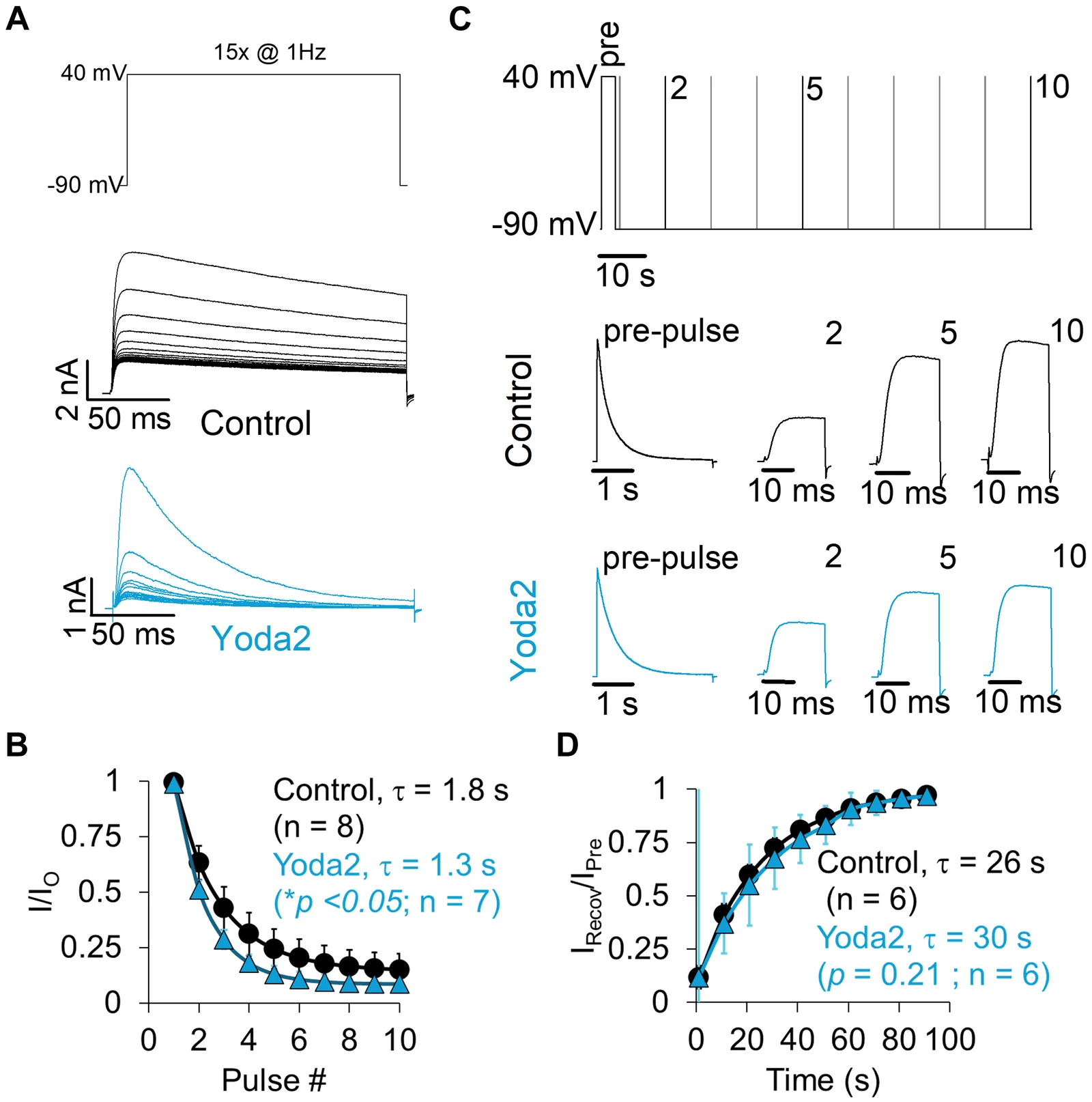
Yoda2 accelerates use-dependent rundown without affecting recovery from inactivation. (A) Protocol schematic (top) and representative current traces at 1 Hz for control (middle) and 30 *μ*M Yoda2 (bottom). (B) Normalized peak current plot demonstrating accelerated use-dependent rundown in the presence of Yoda2 (∗*P* < .05). (C) Recovery from inactivation protocol (top) and representative traces showing the conditioning prepulses and test pulses at varying intervals. (D) Recovery plot of normalized test pulses showing that Yoda2 does not affect recovery kinetics from C-type inactivation (*P* = .21). Data are presented as mean ± SD of *n* ≥6 cells from *N* ≥ 2 independent cultures. Statistical significance was determined by an unpaired Student’s *t* test.

### 3.5 State-independent block and functional rescue of inactivation

While Yoda2 promotes C-type inactivation, its inhibitory activity does not require the channel to be in an open or inactivated state. We confirmed this state-independent mechanism by demonstrating significant resting inhibition even when the channel was held at a hyperpolarized potential (Fig. 3). To further investigate the structural basis of this inhibition, we tested Yoda2 against the inactivation-deficient Kv1.3 H399V mutant; this residue corresponds to H451 in the full-length isoform and is referred to as H451V hereafter.^35^^,^^36^

Consistent with a state-independent mechanism, Yoda2 inhibited the H451V mutant with a potency similar to the WT channel (IC_50_ 6.8 *μ*M, 95% CI, 6.2–7.6 *μ*M; *nH* of 2.2, 95% CI, 1.6–2.9) (Fig. 5, A and B). This behavior contrasts sharply with classic state-dependent Kv1.3 blockers, such as PAP-1 and CP-339818, which exhibit drastically reduced potency on inactivation-deficient mutants because they rely on the open/inactivated state for high-affinity binding to mutants.^23^^,^^37^^,^^38^

**Fig. 5:**
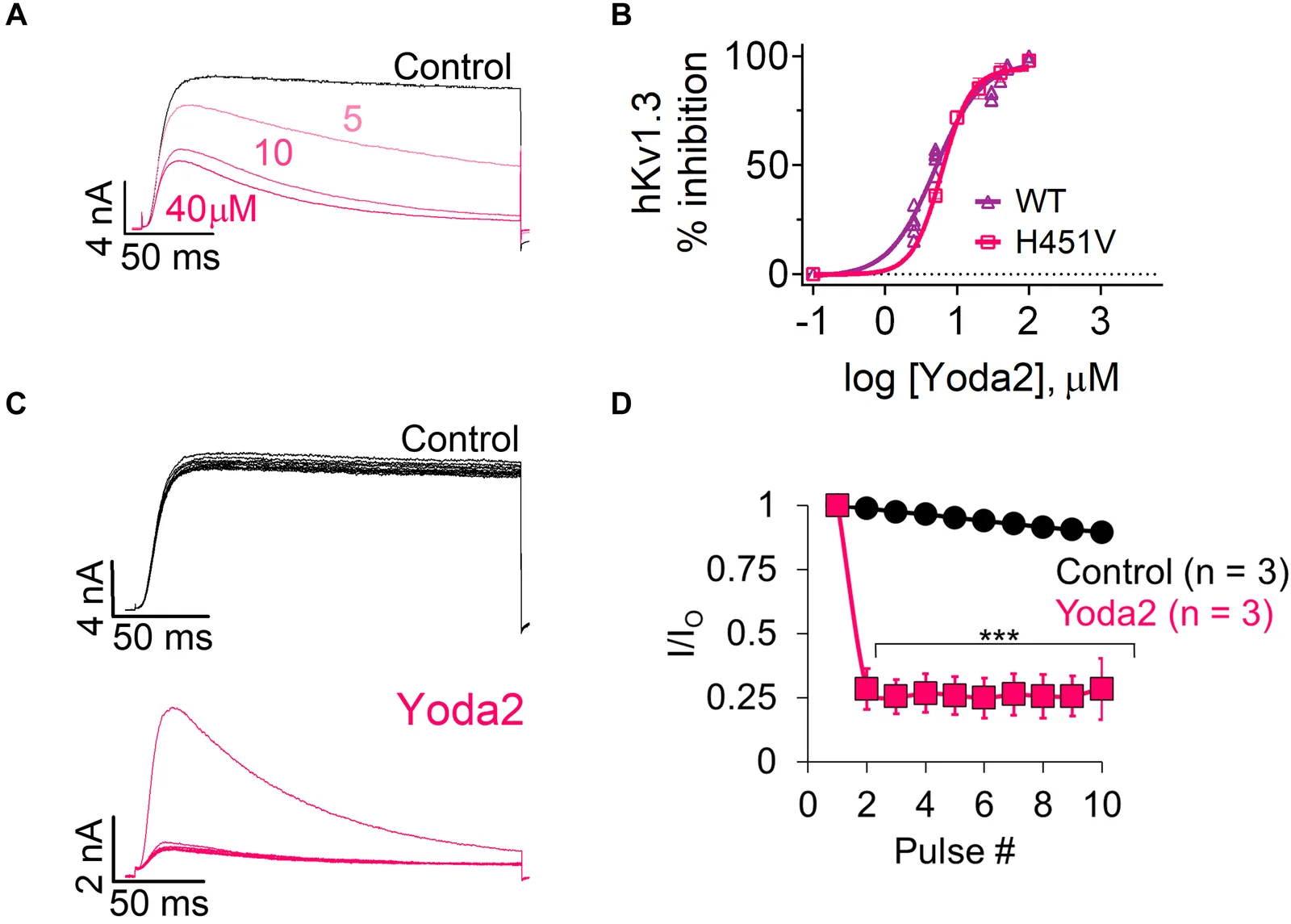
Yoda2 induces cumulative inhibition in inactivation-deficient Kv1.3 H451V mutant. (A, B) Representative current traces (A) and concentration-response curves (B) for Yoda2 against WT and Kv1.3-H451V IC_50_ values (mean, 95% CI): WT: 5.9 *μ*M (5.0–8.6, *n* = 7), H451V, 6.8 *μ*M (6.2–7.6), *n* = 4. (C, D) Use-dependent current traces (1 Hz) for Kv1.3-H451V under control (left, black) and 30 *μ*M Yoda2 (right, blue) conditions. (D) Normalized peak current plot demonstrating that Yoda2 induces cumulative rundown in the otherwise inactivation-deficient mutant. Data are presented as mean and SD. Statistical significance in panel (D) was determined using a paired Student’s *t* test (∗∗∗*P* < .001, *n* = 3). All cells (*n* = 3–7) were recorded from *N* >2 independent cultures.

Intriguingly, although the H451V mutation typically prevents C-type inactivation,^33^ the application of Yoda2 restored a use-dependent current reduction in this mutant (Fig. 5, C and D). This suggests that Yoda2 does not merely wait for the channel to inactivate. Rather, it actively forces the pore into a C-type inactivated conformation, a state the H451V mutant is otherwise resistant to entering. These findings, taken together with the acceleration of kinetics observed in WT channels (Fig. 2), suggest a dual-action mechanism: Yoda2 binds to the channel in the resting state and, upon activation, significantly accelerates the transition into the closed-inactivated state.

To further validate this “rescue” of inactivation, we extended our analysis to other Kv1 family members (hKv1.1, hKv1.2, and hKv1.5), which share high sequence identity in the pore region (Fig. 6A) but lack prominent C-type inactivation. Crucially, Yoda2 not only induced and accelerated rapid inactivation kinetics in these otherwise minimally inactivating channels but also inhibited all 3 related isoforms: hKv1.1 (IC_50_: 8.8 *μ*M, 95% CI, 0.2–17.4 *μ*M; *nH* of 2.2, 95% CI, 0.6–3.8), mKv1.2 (IC_50_: 11.2 *μ*M, 95% CI, 8.2–14.3 *μ*M; *nH* of 2.2, 95% CI, 1.4–2.9), and hKv1.5 (IC_50_: 10.8 *μ*M, 95% CI, 8.5–13.0 *μ*M; *nH* of 1.4, 95% CI, 0.8–2.2). (Fig. 6, B–E). Collectively, the ability of Yoda2 to functionally re-engage the inactivation machinery in both the H451V mutant and confer inactivation in related Kv1 isoforms provides strong evidence that its interaction is mediated by the conserved pore region.

**Fig. 6:**
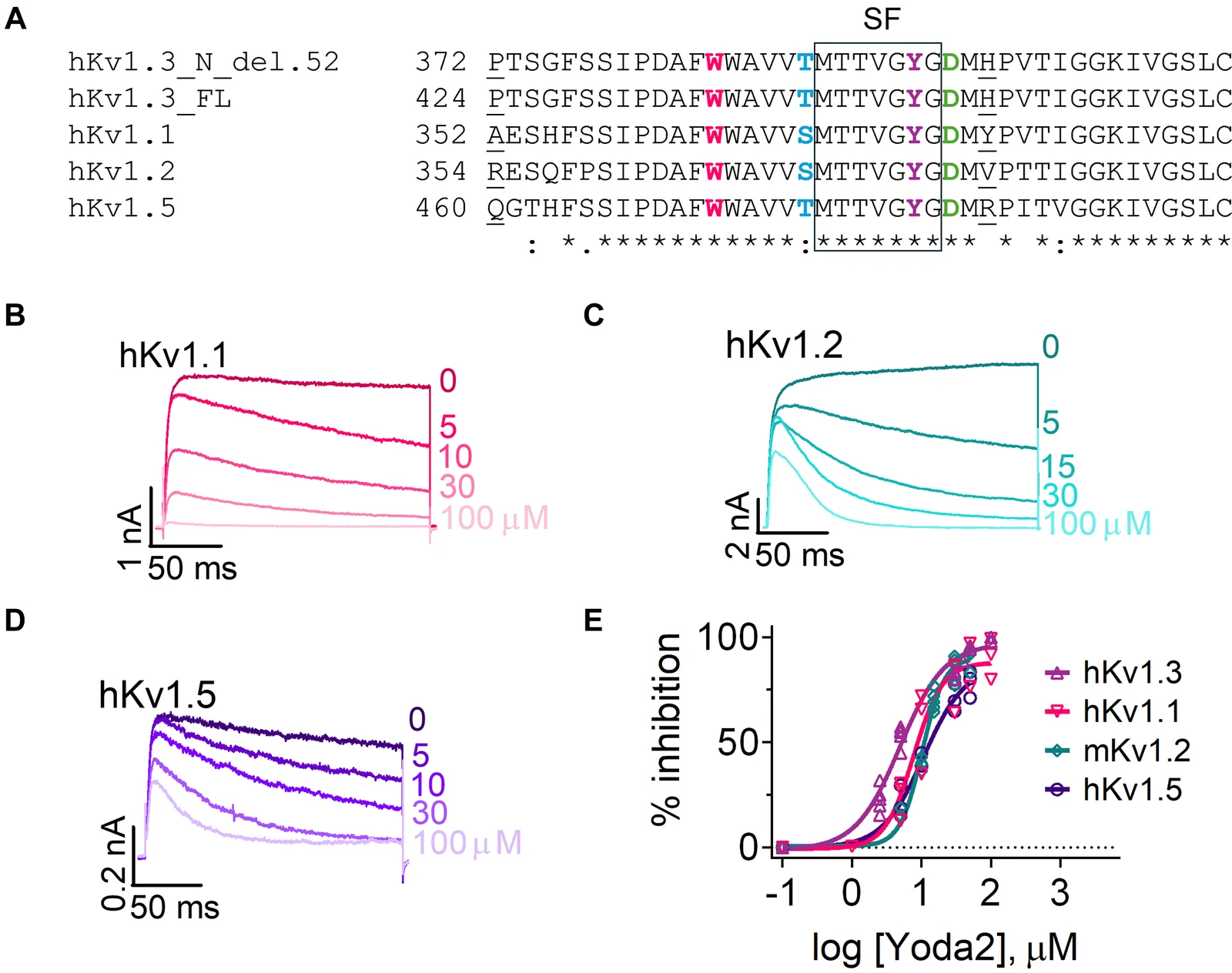
Yoda2 inhibits closely related Kv1-family channels. (A) Sequence alignment of the outer pore region of hKv1.3 (N-terminally truncated [AAC31761.1] and full-length [NP_002223.3]), hKv1.1 (NP_000208.2), hKv1.2 (NP_004965.1), and hKv1.5 (NP_002225.2). Representative current traces showing concentration-dependent inhibition by Yoda2 for (B) hKv1.1, (C) hKv1.2, and (D) hKv1.5. (E) Concentration-response curves and derived IC_50_ values for Kv1.1, Kv1.2, and Kv1.5 compared with hKv1.3. Data are presented as mean ± SD from *n* = 3–5 cells per concentration, with each cell exposed to multiple concentrations to determine the area under the curve (AUC). All cells were recorded from at least *N* = 2 independent cultures.

### 3.6 Binding site prediction for Yoda1 and Yoda2

We next applied molecular docking using the RosettaLigand software to predict the binding sites of Yoda1 and Yoda2 on Kv1.3. Given that these compounds enhance C-type inactivation, our goal was to dock them onto the outer pore region of the channel to capture interactions that mediate the inactivation kinetics observed during our patch-clamp experiments. We thus selected the cryogenic electron microscopic (cryo-EM) structure of the apo human Kv1.3-Kvbeta channel in the C-type inactivated state (Protein Data Bank ID: 7WF3).^39^ This structure was chosen because it is suggested to represent the fully inactivated state, contains the Kv*β* auxiliary subunit, and is free of large modulatory ligands such as nanobodies or ShK-Fab fusion proteins, which could sterically bias the binding pocket.^39^^,^^40^ This inactivated conformation is predicted to result from the breakage of 2 key stabilizing hydrogen bonds, between intrasubunit W436-D449 and intersubunit Y447-T441, which facilitates the outward swing of the Y447 and D449 side chains away from the pore axis. This wide, nonconducting conformation is then secured by a new stabilizing hydrogen bond network between Y447 and D449 with H451, resulting in a dilated, nonconducting pore.^39^^,^^40^

Our docking analysis showed that Yoda1 and Yoda2 converged on a common binding pocket adjacent to the extracellular pore, between 2 adjacent subunits and behind the selectivity filter (Fig. 7A). Despite displaying subtle differences in bonding partners because of the switch from Yoda1’s pyrazine ring to Yoda2’s 4-benzoate to improve solubility,^28^ Yoda1 and Yoda2 adopted a similar orientation, with these groups pointing downwards (Fig. 7, A, C, E, and F). For Yoda1, 9 out of 10 poses converged. The predicted best pose shows that Yoda1 engages in extensive van der Waals interactions with W436, V440, Y447, D449, and H451 from one subunit and Y447, D449, G448, and M450 from an adjacent subunit. In this pose, its pyrazine ring forms 2 hydrogen bonds, one with W436 from one subunit and the second with G448 from another (Fig. 7C). For Yoda2, 8 out of 10 poses converged, with 2 possible subposes. These subposes exhibited differing hydrogen-bonding partners with the same subunit: subpose 1 showed the benzoic acid oxygen hydrogen bonding with Y447, while subpose 2 revealed hydrogen bonds slightly higher up with G488. Both subposes maintained van der Waals interactions with W436, Y447, and D449 from one subunit and Y447, D449, and M450 from the adjacent subunit (Fig. 7, E and F). Overall, despite slight differences in interacting partners between the 2, Yoda1 and Yoda2 showed extensive interactions with key residues known to regulate C-type inactivation, such as W436, D449, and Y447.

**Fig. 7:**
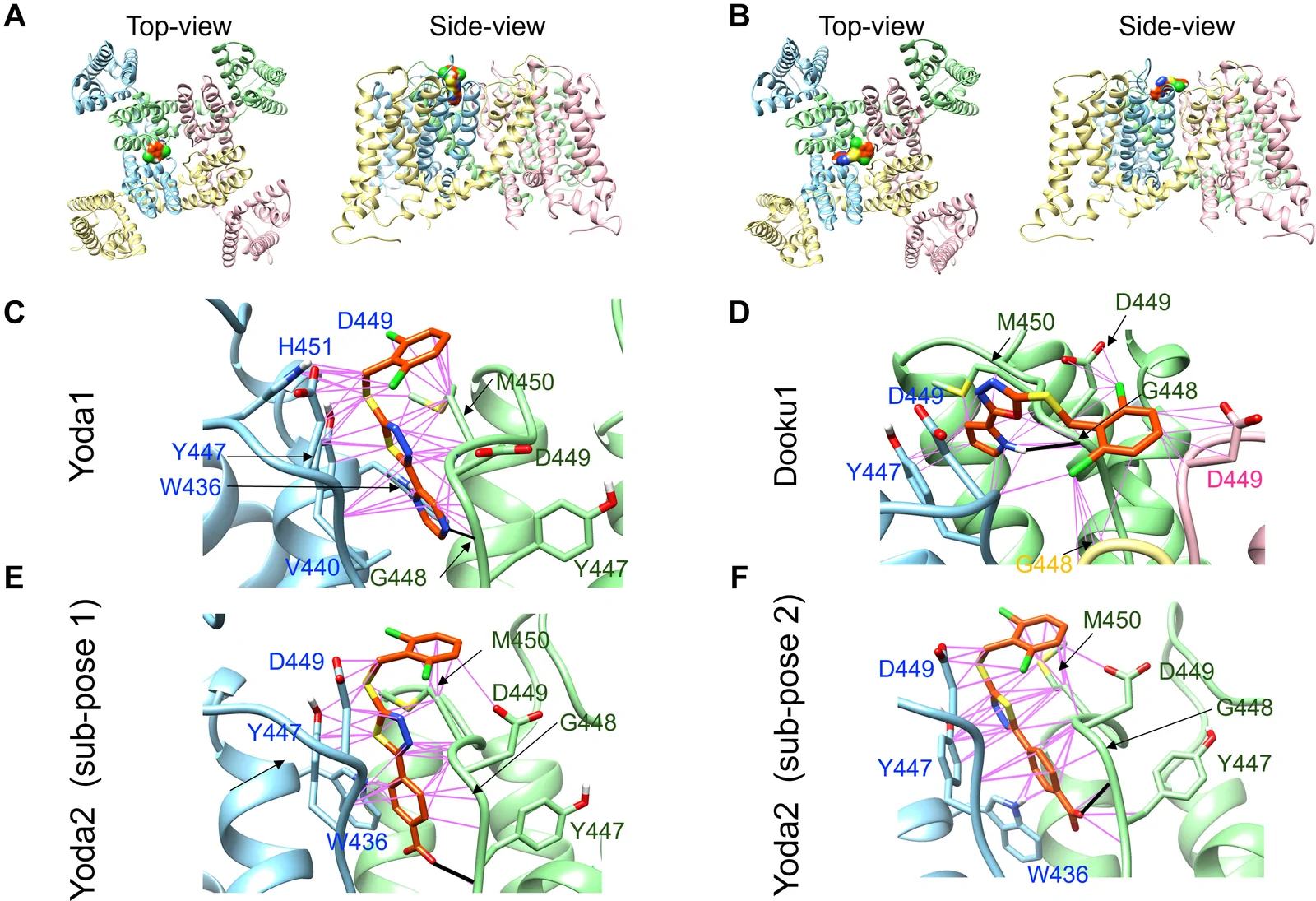
Structural modeling of Yoda compounds docking to human Kv1.3. (A, B) Top- (extracellular) and side-views of the predicted docking poses for (A) Yoda1 and Yoda2, and (B) Dooku1, within the C-type inactivated apo human Kv1.3-Kvbeta channel complex (Protein Data Bank ID: 7WF3). The 4 pore-forming subunits are individually colored for clarity. (C–F) Detailed views of predicted binding interactions for (C) Yoda1, (D) Dooku1, (E) Yoda2 subpose 1, and (F) Yoda2 subpose 2. Hydrogen bonds are depicted as solid black lines and Van der Waals contacts as pink lines. Interacting residues (including W436, V440, Y447, G448, D449, M450, and H451) are highlighted and colored according to their respective subunits.

In contrast, Dooku1, which is inactive on Kv1.3, showed less robust convergence (only 6 out of 10 poses converged). Laying over the pore, Dooku1 was predicted to form a hydrogen bond between its pyrrol-2-yl group and residue G448 (Fig. 7D) while making van der Waals interactions with all 4 channel subunits. Importantly, however, Dooku1 is predicted to assume a much more superficial position compared with Yoda1 and Yoda2, with its pyrrol-2-yl group and an oxadiazole almost horizontal to its dichlorophenyl ring. This surface position might prevent Dooku1 from exerting modulatory effects on channel function.

### 3.7 MD simulations

To evaluate the stability and interaction patterns of the docked ligands, we performed MD simulations and analyzed ligand dynamics and residue-level interactions in the Kv1.3 outer pore region (Fig. 8). Ligand root mean square deviation (RMSD) profiles revealed clear differences in binding stability among the compounds. Yoda1 and Yoda2 maintained relatively low and stable RMSD values across all replicas (Fig. 8, C and D), indicating well defined and stable binding conformations. In contrast, Dooku1 showed significantly higher RMSD values and larger fluctuations (Fig. 8E), suggesting reduced stability of the binding pose. Consistent with these observations, distance analyses between the ligands and key residues (W436, Y447, G448, D449, and H451) showed that Yoda1 and Yoda2 maintained shorter and more stable distances throughout the simulations, whereas Dooku1 displayed greater variability and increased separation from these residues (Supplemental Fig. 2, A–E). Residue–ligand contact frequency further supported these trends: Yoda1 and Yoda2 showed consistently high contact frequencies across all residues, indicating stable interaction with the binding site, while Dooku1 showed weaker and more variable contacts (Supplemental Fig. 2F).

**Fig. 8:**
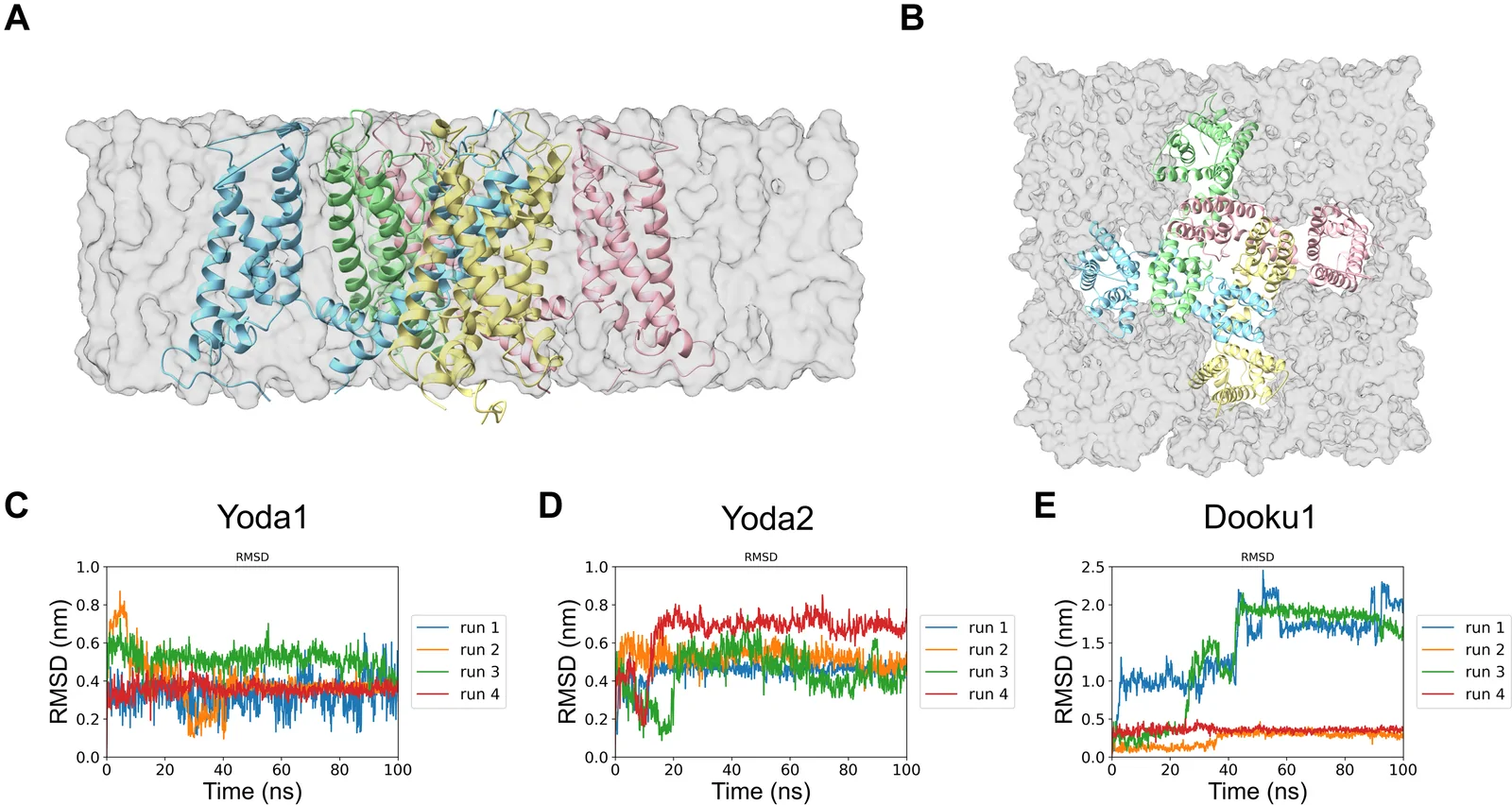
MD simulation analysis of ligand stability in the Kv1.3 outer pore region. (A) Side-view and (B) top-view show the Kv1.3 channel embedded in a membrane environment, providing structural context for the system. Bottom panels display ligand RMSD over time from 4 independent MD simulation replicas for (C) Yoda1, (D) Yoda2, and (E) Dooku1.

Analysis of hydrogen bond interactions showed distinct structural differences between the ligands. Yoda2 displayed higher hydrogen bond frequency and persistence, particularly at residues Y447 and G448, forming stable interactions that support binding stability (Supplemental Fig. 2, G and H). In contrast, Yoda1 showed relatively low hydrogen bond frequency and minimal persistence, suggesting that its binding is not mainly determined by hydrogen bonding. Dooku1 showed moderate hydrogen bond formation; however, these interactions were temporary and did not result in stable binding (Supplemental Fig. 2, G and H). Overall, these results indicate that stable binding in the outer pore region is associated with stable residue contacts rather than hydrogen bond frequency alone. Yoda1 and Yoda2 achieve stable interaction through stable contacts, with Yoda2 additionally benefiting from long-lived hydrogen bond interactions. In contrast, Dooku1 fails to maintain stable interactions, consistent with its reduced binding stability.

### 3.8 Binding site validation

To validate our docking model and MD simulations, we initially performed site-directed mutagenesis on residues identified by RosettaLigand to interact with Yoda1 and Yoda2, specifically focusing on the outer pore and selectivity filter. One such residue, W436, is reported to be essential for functional ion conductance as it forms a critical intrasubunit hydrogen bond with D449 that stabilizes the selectivity filter relative to the pore helix.^41^ Consistent with this structural importance, our experimental attempts to record from W436F, W436A, and W436L, and W436F mutants resulted in nonconducting channels (data not shown). Similar loss-of-function (LOF) was observed for mutations at Y447 (F), G448 (A and S), and D449 (E and N). The M450L substitution was the only mutant that successfully yielded functional currents, but it exhibited significantly altered baseline kinetic signatures (Supplemental Fig. 3). While these results preclude us from accurately measuring the leftward or rightward shifts in Yoda2 potency, they confirm that the predicted binding pocket is situated within a high-sensitivity structural “hotspot” essential for pore architecture.^41^, ^42^, ^43^, ^44^, ^45^, ^46^

### 3.9 Yoda2 inhibits Kv1.3’s regulation of membrane potential

Because Kv1.3 activity is crucial for repolarizing the membrane potential by mediating K^+^ efflux, we sought to investigate whether Yoda2 would impair the ability of Kv1.3 to prevent membrane depolarization. To test this, we first used Kv1.3-expressing CHO stable cells. In control cells, a current injection ramp protocol induced minimal membrane depolarization, demonstrating Kv1.3’s robust buffering capacity (Fig. 9, A and C). However, in the presence of Yoda2, the same current injections induced massive membrane depolarization (Fig. 9, B and C), indicating that it impaired Kv1.3’s ability to buffer extreme membrane depolarization. We further confirmed Yoda2’s inhibition of native Kv1.3 currents in tissue macrophages^19^^,^^47^ and the resulting impact on membrane potential regulation in ex vivo, acutely isolated cardiac tissue macrophages stimulated with LPS for 24 hours (Fig. 9, D–F).

**Fig. 9:**
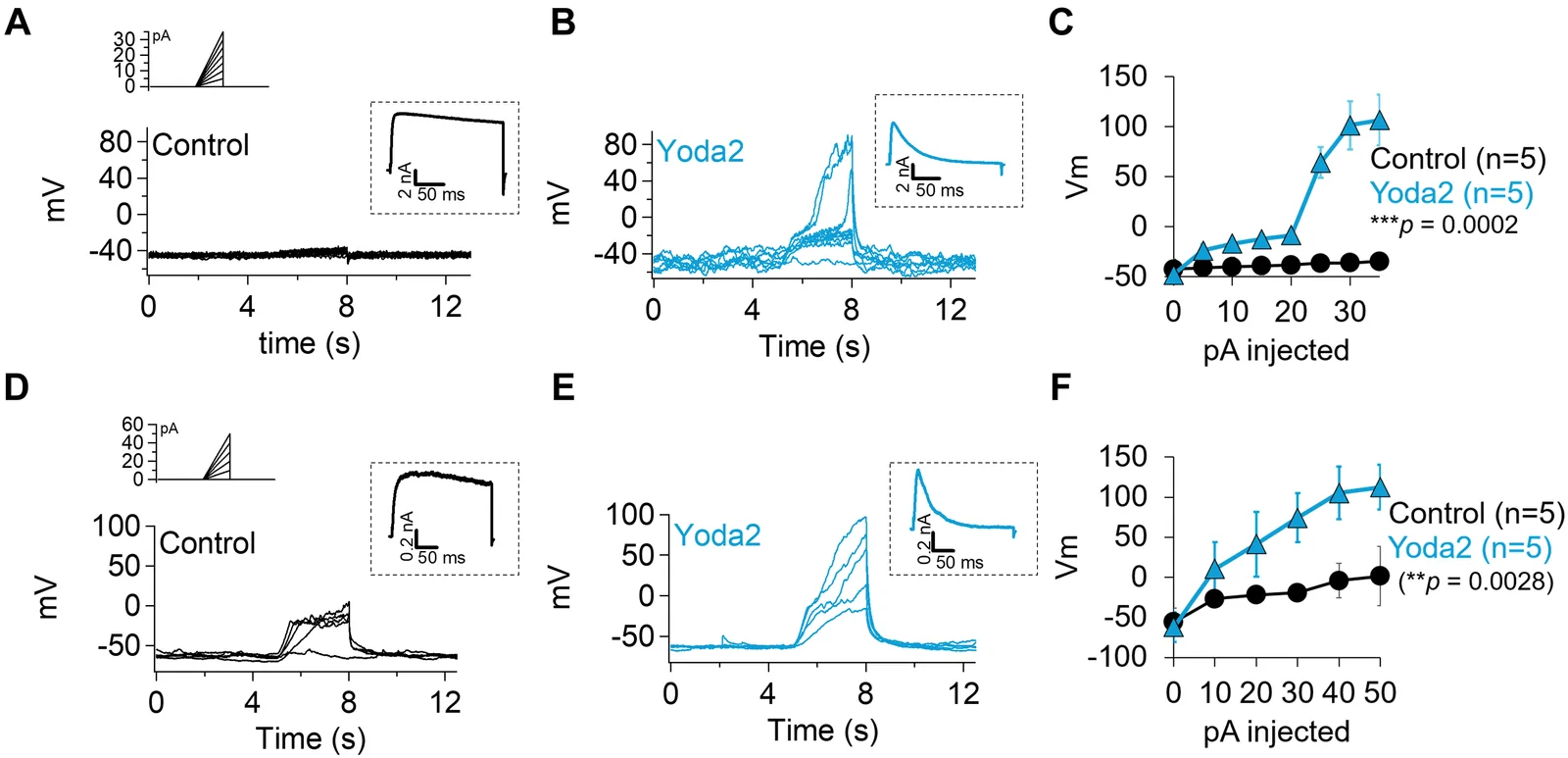
Yoda2 prevents Kv1.3-mediated membrane repolarization in CHO cells and macrophages. Yoda2 prevents Kv1.3-mediated membrane repolarization in CHO cells and macrophages. (A, B) Representative current-clamp traces from a CHO cell expressing hKv1.3 during current ramp injections (top inset) recorded under (A) control conditions and (B) following 30 *μ*M Yoda2 application within the same cell. (C) Quantification of peak membrane potential measured at the end of current injection steps (*n* = 5 cells nested within *N* = 2 independent biological replicates; ∗∗∗*P* = .0002 by nested *t* test). Data are mean ± SD. (D, E) Representative traces from an LPS-activated macrophage (250 ng/mL, 24 hours) showing membrane potential (D) before and (E) after 30 *μ*M Yoda2 treatment. (F) Quantification of peak membrane potential in macrophages (*n* = 5 cells nested within *N* = 2 independent biological replicates; ∗∗*P* = .0028 by nested *t* test). Data presented as mean ± SD.

### 3.10 Yoda2 prevents store-operated calcium entry and IL-1β production in Piezo1-deficient BMDM

Because Yoda2, via blocking Kv1.3, prevents cellular repolarization, it is expected to have a direct effect on the electrochemical driving force for Ca^2+^ entry from the extracellular space. To enable direct measurement of Yoda2’s effect on Kv1.3’s regulation of Ca^2+^, we differentiated bone marrow cells from a LOF Piezo1 transgenic mouse line expressing the genetic Ca^2+^ sensor Salsa6 under the Cx3cr1 promoter into BMDM (PZ1LOF_BMDM) through culturing with MCSF for 5 days, followed by MCSF and granulocyte-MCSF for 2 days.^27^ In vitro differentiated PZ1LOF BMDMs showed upregulated Kv1.3 expression upon stimulation with LPS (Fig. 10, B, C, and E), but did not further increase cell size, as cell capacitance did not change (Fig. 10D). The latter is likely a result of the inclusion of MCSF during the differentiation process from monocytes into macrophages. LPS-induced Kv1.3 currents in these reporter BMDM were sensitive to inhibition by Yoda2 in a similar dose-dependent manner as recombinant Kv1.3 (Fig. 10F). Yoda2 did not trigger increases in intracellular Ca^2+^, confirming the loss of Piezo1 expression (Fig. 10A). However, Yoda2 significantly reduced store-operated calcium entry (SOCE) mediated through CRAC channels, consistent with the effect of PAP-1, a known Kv1.3 inhibitor, on CRAC-mediated Ca^2+^ influx^22^ (Fig. 10, G and H; *P* < .001).

**Fig. 10:**
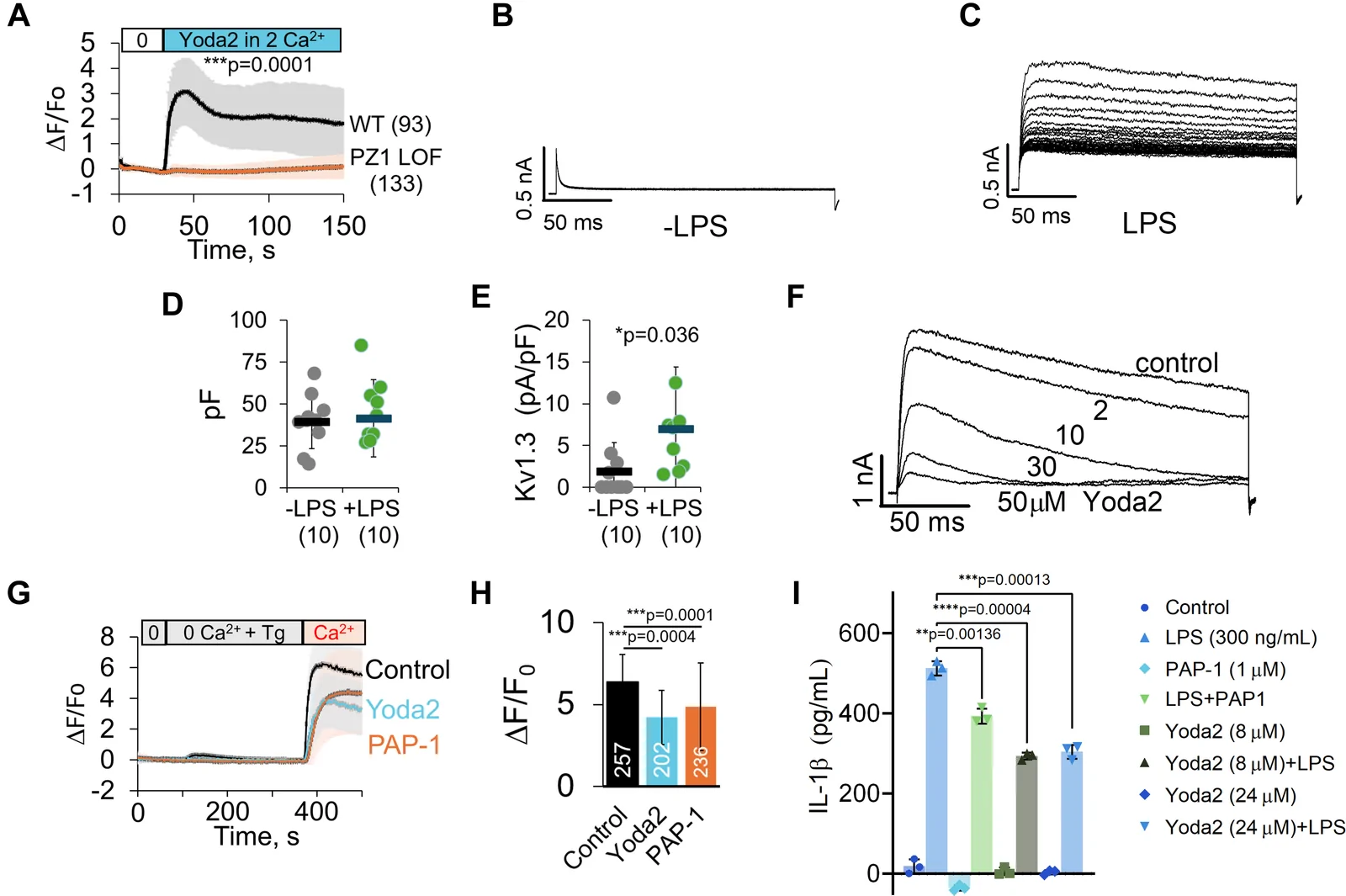
Yoda2 inhibits SOCE and IL-1*β* secretion in Piezo1-deficient BMDMs. (A) Ca^2+^ imaging of WT and Piezo1 LOF BMDMs showing the absence of Yoda2-induced (30 *μ*M) Ca^2+^ influx in LOF cells (*n* ≥93 cells nested within *N* = 3). (B, C) Representative voltage-clamp traces from (B) unstimulated and (C) LPS-treated (250 ng/mL, 24 hours) LOF BMDMs. (D, E) Summary of (D) cell capacitance and (E) Kv1.3 current density (*n* = 10 cells, *N* ≥3). (F) Representative traces from a LOF cell showing dose-dependent inhibition of Kv1.3 by Yoda2 (1–30 *μ*M). (G) Ca^2+^ imaging traces showing SOCE inhibition by 30 *μ*M Yoda2 or 1 *μ*M PAP-1. (H) Peak SOCE quantification (*n* ≥ 202 cells nested within *N* = 3). (I) ELISA of IL-1*β* release induced by LPS (300 ng/mL, 24 hours) comparing control to Yoda2 and PAP-1 treatments (*N* = 3; dots represent means of technical triplicates). Data are presented as mean ± SD. Statistical significance was determined by a nested *t* test for multilevel multiple comparisons against control/LPS-only groups.

Ca^2+^ is an important regulator of inflammatory pathways, including the synthesis and release of many proinflammatory cytokines. Inhibitors of Kv1.3 are known to prevent the expression of IL-1*β*. Stimulation of PZ1-LOF BMDM with LPS resulted in a massive increase in the release of IL-1*β* as measured by ELISA. Yoda2 treatment reduced supernatant IL-1*β* levels (Fig. 10I), similar to the Kv1.3 blocker, PAP-1.

## 4 Discussion

Macrophages and microglia dynamically shift between inflammatory and reparative states in response to changes in their environment.^48^^,^^49^ Piezo1 drives immune responses in macrophages and microglia by transducing mechanical forces into intracellular Ca^2+^ signaling.^6^^,^^7^^,^^50^, ^51^, ^52^ Consistent with its proinflammatory role, Piezo1 activation, whether by physical forces or by its pharmacological agonist Yoda1, has been widely reported to enhance inflammatory responses and increase the release of cytokines such as IL-1*β*, IL-6, and TNF-*α*.^10^^,^^12^ Conversely, Piezo1 inhibition or deficiency reduces cytokine production and suppresses Nod-like receptor protein 3 and NF-*κ*B activation.^12^^,^^13^

Although Piezo1 is largely considered proinflammatory, its pharmacological potential remains obscured by conflicting reports that its selective agonist, Yoda1, can also inhibit inflammatory responses. For instance, Yoda1 was shown to inhibit NF-*κ*B activation and the release of TNF-*α* and IL-6 in various innate immune cell types.^11^^,^^14^ Furthermore, in neurodegenerative models, Piezo1 activation with Yoda1 reduced neuroglial inflammation, A*β* deposition, and tau hyperphosphorylation in a microfluidic model, alleviating synaptic impairment and neuronal loss.^15^ This perspective is supported by findings that treatment with Yoda1 ameliorates A*β* burden and cognitive impairment in 5 Familial Alzheimer's Disease mutations (5xFAD) mice.^53^^,^^54^

These anti-inflammatory outcomes present a biological paradox as increased intracellular Ca^2+^ is widely recognized as a mediator of inflammatory pathway activation. Specifically, the key inflammatory transcription factor, NF-*κ*B, is Ca^2+^-dependent, with elevated intracellular Ca^2+^ enhancing its activity.^55^, ^56^, ^57^ Therefore, reports of anti-inflammatory effects of Yoda1, which promotes Ca^2+^-conducting activity by reducing the mechanical activation threshold of Piezo1, warrant a deeper investigation into possible off-target effects or unknown mechanisms. Our data demonstrate that Yoda1 and its more soluble derivative, Yoda2, block Kv1.3 currents. Critically, Yoda2 depolarizes the membrane and reduces store-operated calcium entry–mediated Ca^2+^ signaling and IL-1*β* release in Piezo1-deficient BMDMs. By inhibiting Kv1.3, a key regulator of RMP, these agonists dissipate the electrochemical gradient, thereby suppressing the Ca^2+^ driving force essential for immune cell activation.^16^^,^^17^^,^^22^

This mechanism specifically targets activated immune cells, which explains why Yoda1-mediated anti-inflammatory effects are only observed under stimulation (eg, LPS or A*β*) rather than in basal states.^10^, ^11^, ^12^^,^^14^^,^^53^^,^^54^ Because Kv1.3 is significantly upregulated in activated macrophages, microglia, and AD models, its blockade becomes a dominant physiological factor during inflammation.^20^^,^^21^^,^^25^^,^^47^ Given that Kv1.3 inhibition is a well established anti-inflammatory pathway,^22^^,^^25^ we propose that the reported clinical benefits of Yoda1 and Yoda2 in macrophages and microglia^11^^,^^14^ are significantly mediated by off-target Kv1.3 blockade, in addition to, or potentially independent of, canonical Piezo1.

Yoda1 and Yoda2 inhibit Kv1.3 within the same low-micromolar functional window (IC_50_ = 5.9 *μ*M for hKv1.3) routinely used to study Piezo1, where automated patch-clamp benchmarks establish agonist EC_50_ values between 1.5 and 1.7 *μ*M.^28^^,^^58^ This “off-target” overlap is highly significant, as these agonists are typically deployed, for example, at 0.1–10 *μ*M to investigate Piezo1-mediated signaling in vascular migration or clinical indications such as lymphedema.^59^ Notably, in neurodegeneration research, concentrations reaching 20 *μ*M are used to stimulate microglial plaque clearance, a range in which our results suggest Kv1.3 would be nearly fully inhibited, potentially confounding the interpretation of Piezo1-specific effects in these immune cells.^60^

Despite this potency overlap, several lines of evidence suggest that standalone Piezo1 activity did not contribute to our observations. Although Syeda et al^8^ reported robust Piezo1-mediated Ca^2+^ flux at these concentrations, they also found that 10 *μ*M Yoda1 failed to elicit measurable currents in whole-cell patch-clamp recordings without mechanical force. We further confirmed a direct mechanism by demonstrating that Yoda2’s inhibitory effect remains intact in Piezo1-KO HEK-293 cells and BMDMs from Piezo1-KO mice, even with 10 mM intracellular BAPTA to eliminate Ca^2+^-mediated signaling. While these pharmacological and genetic controls rule out indirect modulation, future biophysical studies, such as cryo-EM or site-specific photoaffinity labeling, will be essential to definitively validate the physical binding coordinates predicted by our docking data.

While their inhibition of Kv1.3 is state-independent, both Yoda1 and Yoda2 accelerated Kv1.3’s C-type inactivation. Kv1.3 is biophysically characterized by its rapid activation followed by relatively slow C-type inactivation, similar to that of other Kv channels and the Shaker K^+^ channel, and pronounced cumulative inactivation.^33^^,^^41^^,^^61^, ^62^, ^63^, ^64^, ^65^ Recent cryo-EM structures of the human Kv1.3 channel in the open-inactivated state reveal a drastically restructured and dilated selectivity filter.^39^^,^^40^ The mechanism underlying the transition from the activated open to the inactivated conformation is suggested to involve the breakage of 2 key stabilizing hydrogen bonds, between intrasubunit W436-D449 and intersubunit Y447-T441, which facilitates the outward swing of the Y447 and D449 side chains away from the pore axis. This wide, nonconducting conformation is then locked by a new stabilizing hydrogen bond network consisting of Y447-H451 and D449-H451, resulting in a dilated and nonconducting pore because of the loss of K^+^ coordination.^34^^,^^39^^,^^40^

Our docking analysis revealed that Yoda1 and Yoda2 converge on a common binding pocket, adopting a shared orientation that points deep into the selectivity filter and wedges between 2 adjacent channel subunits. Both are predicted to interact with key residues (W436, D449, and Y447) known to regulate C-type inactivation. We propose that Yoda1 and Yoda2 destabilize crucial intrasubunit (W436-D449) and intersubunit (Y447-T441) hydrogen bonds, weakening the structural network that stabilizes the pore and thereby enhancing Kv1.3’s entry into inactivation. Destabilization of the pore by Yoda2 is also supported by our data indicating changes in voltage-dependent properties and enhanced deactivation kinetics.

The H451 residue (H404N in mKv1.3 and H449 in Shaker) in the S5–S6 linker is critical for Kv1.3 inactivation.^34^^,^^37^^,^^62^^,^^66^ Mutations such as H451N accelerate inactivation,^6^^,^^66^ while H451V drastically slows it.^36^ Based on cryo-EM structures, H451 is proposed to reinforce the dilated pore conformation by engaging in a secondary, intersubunit interaction with D449 and Y477, stabilizing the pore after the breakage of the W436-D449 and Y447-T441 bonds.^39^^,^^40^ Not only does Yoda2 inhibit Kv1.3 in a state-independent manner, but it was also still effective in inducing C-type inactivation in the Kv1.3 H451V mutant. This suggests that Yoda2 not only disrupts the pore’s interaction network to initiate pore dilation but also actively stabilizes the dilated pore conformation, negating the need for the canonical stabilizing effect of H451.

## 5 Conclusion

Overall, we have demonstrated that off-target blockade of Kv1.3 contributes significantly to the anti-inflammatory effects attributed to Yoda1 and Yoda2. This mechanism reconciles discrepancies regarding Piezo1 agonism in inflammation, aligning with the principle that reduced intracellular Ca^2+^ is anti-inflammatory, whereas sustained calcium signaling is proinflammatory. Although transient Piezo1-mediated Ca^2+^ influx may assist acute pathogen clearance, continuous activation during chronic neuroinflammation is likely detrimental. Instead, our findings reinforce the therapeutic benefit of blocking Kv1.3, which is upregulated in chronic neuroinflammation, to prevent secondary injury.^20^^,^^21^^,^^67^, ^68^, ^69^

Given our findings, future development should leverage high-resolution cryo-EM structures of Piezo1 and Kv1.3 or comparative molecular docking and virtual screening to identify leads that avoid crossreactivity. Additionally, selectivity screening must prioritize direct electrophysiology over standard calcium or radioligand assays to account for assay-dependent potency shifts. Early integration of these rigorous methods will ensure that unintended voltage-gated channel inhibition, such as the Kv1.3 blockade identified here, is accurately detected.

## Conflict of interest

The authors declare no conflicts of interest.
