# Supplementary material for: Piezo1 agonists are potent off-target inhibitors of the Kv1.3 channel via gating modification
Source: Mol Pharmacol. 2026 Jul 29;108(9):100149. doi: 10.1016/j.molpha.2026.100149 (PMC13688990; doi:10.1016/j.molpha.2026.100149)

MOLPHA-D-26-00018 Supplemental Materials. Huang et al. Mol Pharmacol

Piezo1 Agonists are Potent Off-Target Inhibitors of the Kv1.3 Channel via Gating Modification

Kyan Huang, Heesung Shim, Heike Wulff, Hai M. Nguyen

Department of Pharmacology (K.H., H.W., H.M.N.), School of Medicine, University of California, Davis, CA 95616, USA; Physical and Life Sciences (H.S.), Lawrence Livermore National Laboratory, Livermore, CA 94550, USA

Figure Legends for Protein Data Bank (PDB) Files

PDB #1. Docked model shown predicted binding interactions for Dooku1

PDB #2. Docked model shown predicted binding interactions for Yoda1

PDB #3. Docked model shown predicted binding interactions for Yoda2 subpose #1

PDB #4. Docked model shown predicted binding interactions for Yoda2 subpose #2

Supplemental Figure 1 - Dooku1 is inactive and does not prevent Yoda2 from blocking Kv1.3. Representative current traces of human Kv1.3 in the absence (control) or presence of pre-incubated with 50 μM Dooku1, 50 μM Dooku1+ 20 μM Yoda2, or after washout.

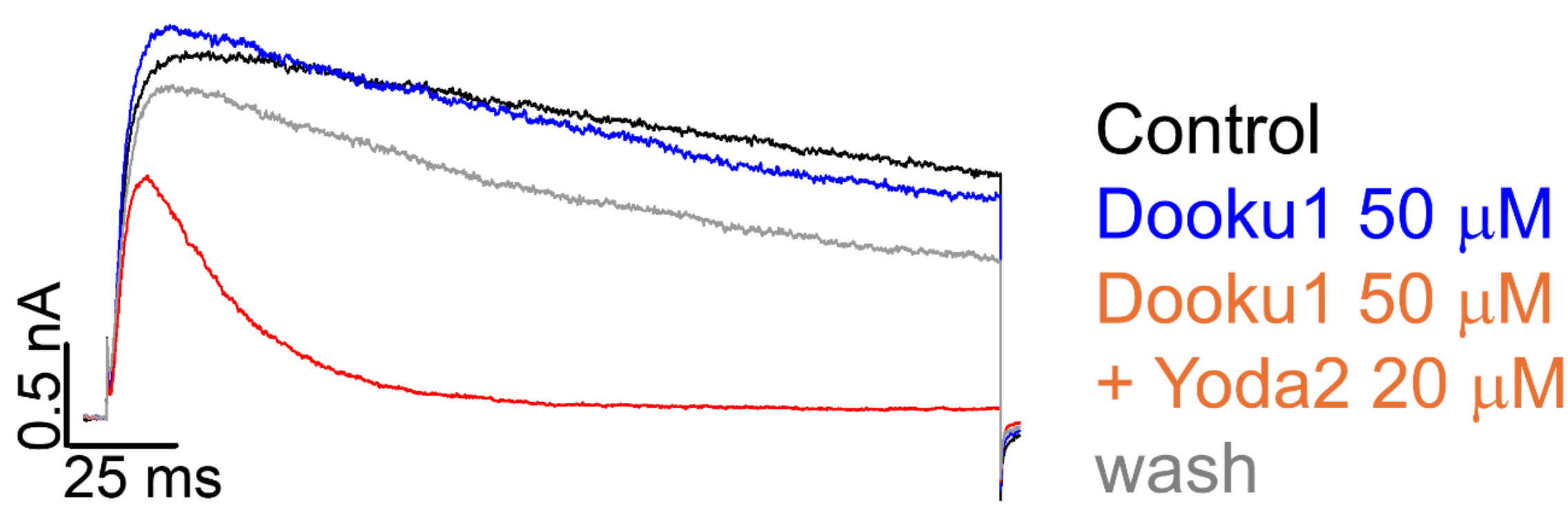

Supplemental Figure 2. MD analysis of ligand–residue interactions in Kv1.3. (A–E) Distance between ligands and key residues over time. (F) Residue–ligand contact frequency, defined as the fraction of simulation time during which each residue remains in contact with the ligand. (G) Hydrogen bond frequency, representing how often hydrogen bonds form between the ligand and key residues during the simulations. (H) Hydrogen bond persistence, defined as the average duration (in frames) of continuous hydrogen bond interactions.

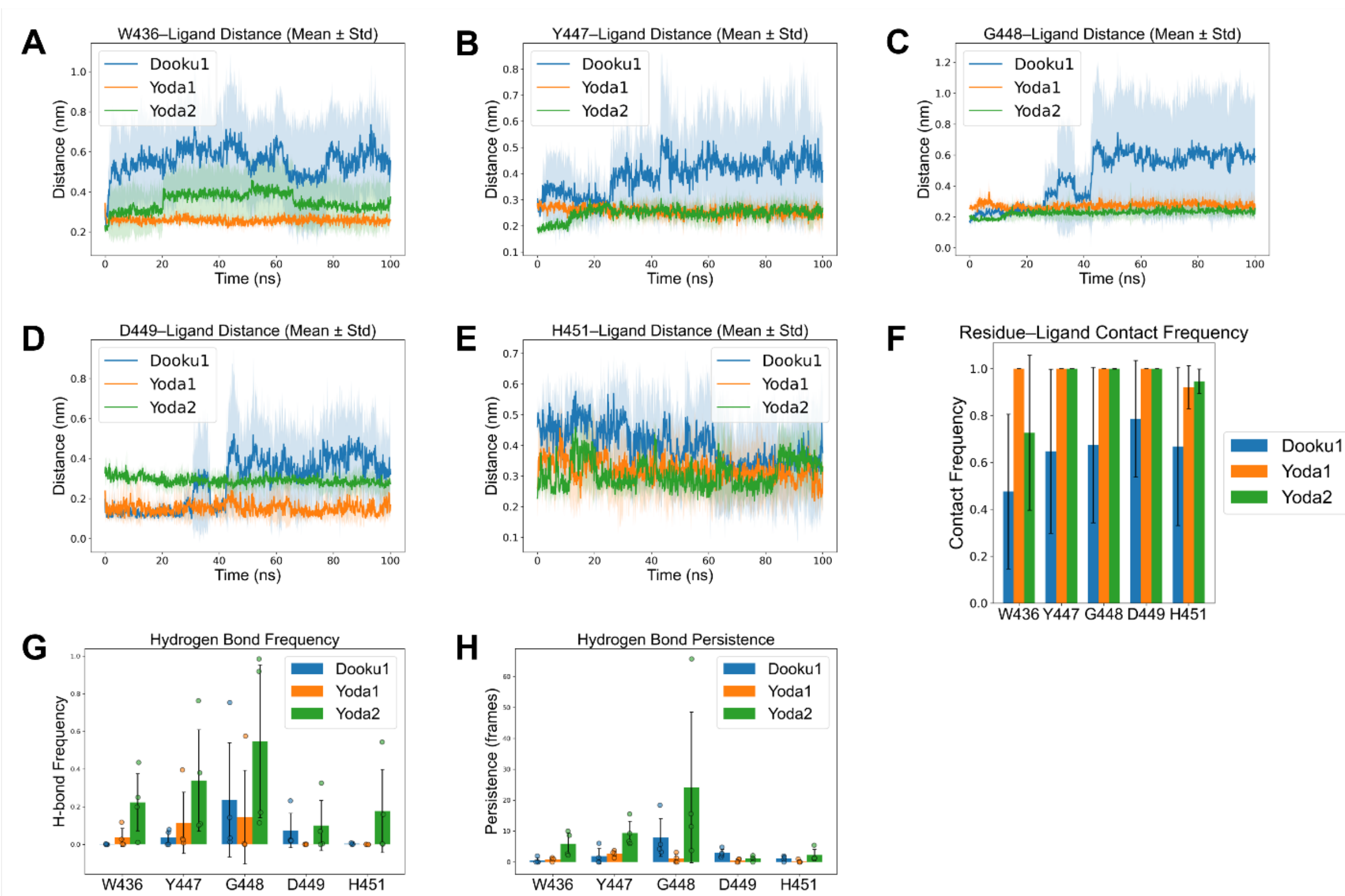

Supplemental Figure 3. The hKv1.3-M450L mutant exhibits accelerated C-type inactivation. (A) sample current trace to +40 mV from -90 mV holding potential. (B) Enhanced use-dependent current decay. (C) Sample current traces show current rundown upon consecutive depolarization at normal interpulse intervals of 30 seconds. (D) Plot showing current rundown.

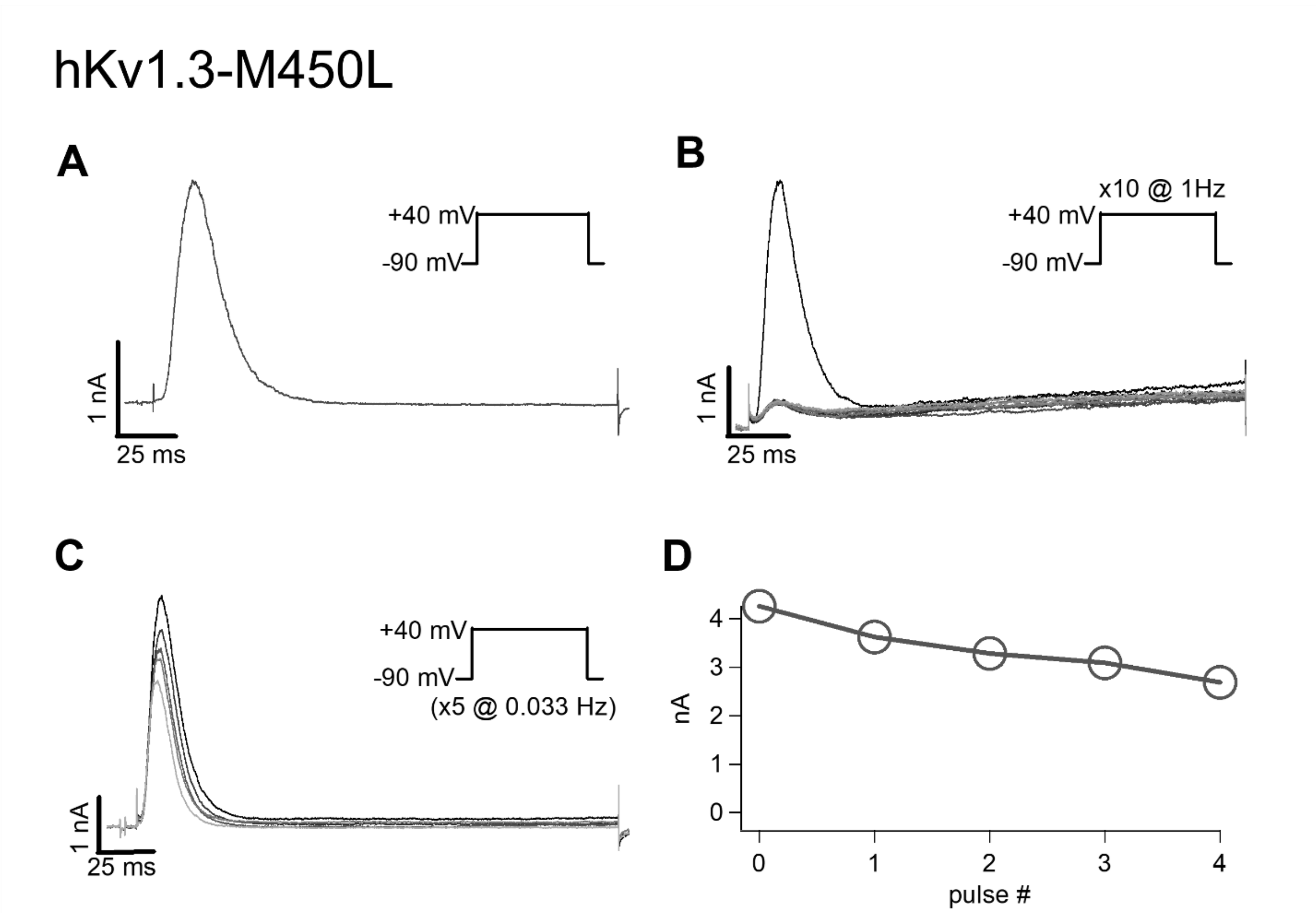

Supplement: Supplementary file 1 [file mmc1.pdf]
